# “There is not much help for mothers like me”: *Parenting Skills for Mothers with Borderline Personality Disorder* – a newly developed group training program

**DOI:** 10.1186/s40479-016-0050-4

**Published:** 2016-12-01

**Authors:** Babette Renneberg, Charlotte Rosenbach

**Affiliations:** Department of Education and Psychology, Clinical Psychology and Psychotherapy, Freie Universität Berlin, Habelschwerdter Allee 45, D - 14195 Berlin, Germany

**Keywords:** Borderline Personality Disorder, Parenting skills, Group training, Motherhood

## Abstract

**Background:**

Dysfunctional relationships and emotion dysregulation are hallmark features of borderline personality disorder (BPD). Women with BPD are, therefore, particularly challenged when raising a child. A group training program was developed for mothers with BPD to enhance their parenting skills and help them raise their children. The program is based on cognitive-behavioral principles and skills derived from Dialectical Behavior Therapy (DBT).

**Methods:**

*N* = 15 mothers with BPD who had young children (aged 0–6 years) participated in a 12-week training program. To estimate the participants’ impairment, parental stress and psychological distress were assessed before the training. After the training, participants and trainers were asked to provide feedback regarding the evaluation of and the changes due to the training.

**Results:**

Participants’ self-reported stress related to parenting, as well as psychological distress and depressive symptoms, was high. Participants’ acceptance of the program was very good. Especially role plays were rated as useful. Trainers evaluated the program as helpful and reported visible changes in participants’ behavior and attitudes towards parenting.

**Conclusions:**

The results on the acceptance of the training program are promising.

**Trial registration:**

NCT02935218, Unique Protocol ID: RenRos01

Initial release 80 August 2016, last release 13 October 2016; ‘retrospectively registered’

## Background

Raising children is a great pleasure and at the same time a big challenge. In the first years of parenting, parents struggle with major adjustments in their lives: they suffer from sleep deprivation, are faced with a change of life focus, must organize the daily routine according to the children’s needs and so forth.

The daily life of women with Borderline Personality Disorder (BPD) is characterized by recurring and frequent changes in mood, self-image, and identity. In stressful situations, the women tend to react with impulsive or self-harming behavior, easily lose their temper, or engage in excessively self-damaging behavior (e.g., drinking, drug use). At the same time, individuals with BPD have difficulties engaging in and especially maintaining stable relationships. This “stable instability” of interpersonal and emotional problems [1] affects all daily interactions, including the parents’ relationship with their infant(s). Taking into account that stability and dependability of the caregiver are crucial for a healthy child development, women with BPD are extremely challenged when expecting and raising a child.

A newly developed group training program for mothers with BPD is described [2]. The program is designed to enhance the mothers’ parenting skills by teaching them about the primary needs of the child, coping with stress, emotion regulation, and self-care.

## Mothers with BPD

The existing literature points to the numerous difficulties mothers with BPD face (for an overview see [3]). Compared to clinical and non-clinical control groups, the level of parental depression is elevated in samples of mothers with BPD; they engage more often in alcohol and drug abuse and show suicidal behavior [4, 5]. Mothers with BPD often have difficulties in accurately perceiving and reacting to their own emotional needs. Therefore, findings demonstrating the difficulty of mothers with BPD to respond sensitively to their infant’s needs are not surprising (e.g. [6]). By analyzing their speech and behavior in the interaction with their offspring, mothers with BPD were characterized as intrusive and insensitive [7]. Compared to a healthy control group, mothers with BPD showed more affective dysregulation in the communication with their children as well as more critical and intrusive behaviors, role confusion, and frightened/frightening behaviors [8]. Additionally, a reduced ability of mothers with BPD to think about their children’s mental state (“mind-mindedness”) compared to healthy controls was observed [9].

Mothers with BPD were more inclined to inhibit autonomy and relatedness in their teenaged children than a group of healthy mothers, whereby this inhibition tendency of the mothers was associated with adolescent internalizing and externalizing symptoms [10]. Regarding mother-infant interaction and parenting perceptions, mothers with BPD reported lower levels of maternal self-esteem, competence and satisfaction, higher distress and less structure in their interaction with their offspring than a healthy control group [6].

In their attachment patterns, mothers with BPD were significantly more likely to be classified as insecure, preoccupied, and unresolved than a normative comparison group [11]. Children’s disruptions in attachment (e.g., fear of abandonment and role reversal) and self-regulation were associated to their mothers’ preoccupied and unresolved attachment style. The significance of role confusion for the development of mental distress in children and adolescents has recently been highlighted by Macfie and colleagues [12].

In addition to the mental health problems of mothers with BPD, there are numerous other stressors, mostly social strains the mothers have to deal with, e.g., being a single parent, having low social support in the family, changing partners, instable households (e.g. [4]).

## Children of mothers with BPD

A mental disorder of one or both parents is a general risk factor for psychological distress in children (e.g. [13]). Children of mothers with BPD have a higher prevalence of emotional and behavioral problems than children of healthy mothers as well as children of mothers with other mental disorders [14–16].

In the following, empirical evidence for the difficulties and problems children of mothers with BPD have is summarized based on the age of the children. Emotional dysregulation starts early in children of mothers with BPD. Using the still-face paradigm, Crandell et al. [17] observed less responsiveness in children of mothers with BPD toward their mother than infants of mothers without a mental disorder. One year later, 80% of this sample showed disorganized attachment behavior towards their mothers [7]. Two studies indicate that the reunion episode of the still-face paradigm seems particularly challenging for the dyads [17, 18]. Mothers showed fewer smiles than mothers of the control group and an increase in intrusive behavior. Their children in turn expressed less positive phonation [18] and more negative affect than infants of mothers with no psychopathology. The children also took longer to reengage with their mothers [17].

In a free-play interaction, infants of mothers with BPD were less attentive and less interested in interactions with their mother compared to children of healthy mothers [6]. In a recent study, maternal BPD symptoms were related to either very high or very low but less to moderate levels of fear in 12- to 23-month-old infants, indicating disturbed levels of children’s fear expression [19].

Regarding psychopathology and mental disorders in school-aged and adolescent children of mothers with BPD, this population showed more disruptive behavior, more Attention Deficit/Hyperactivity Disorder (ADHD) and more borderline symptoms than children of healthy mothers [4, 20]. They tend to have higher levels of anxiety and depression, and significantly lower self-esteem compared to children of healthy mothers and children of mothers with depressive disorder and cluster C personality disorders [15]. In a story completion task, 4–7 year-old children of mothers with BPD showed poorer emotion regulation, more role reversal, greater fear of abandonment, more negative parent-child relationship, as well as more incongruent and shameful self-representations than a healthy control group [21]. In a large community sample, maternal BPD symptoms were associated with poorer psychosocial functioning in adolescents (e.g., lower social self-perception) as well as with higher and chronic stress in the mother-adolescent relationship, and greater maternal hostility [16]. Reinelt and colleagues [22] investigated 15-year old adolescents and found that particularly an overprotective and rejecting parenting style as well as discrepancies in the perception of psychopathological problems of the offspring mediate the longitudinal transmission of borderline symptoms from mother to child 5 years later.

In summary, children of mothers with BPD have a greater risk for emotional and behavioral problems than children of healthy mothers or children of mothers with other mental disorders such as depression. Especially in early childhood, the maternal behavior is crucial for the development of personal needs, attachment patterns, identity, and interpersonal behavioral strategies. We, therefore, aimed to conceptualize a training program for mothers with BPD to enhance their parental skills and facilitate a healthy child development.

## The training program “*Parenting Skills for Mothers with Borderline Personality Disorder*”

*Parenting Skills for Mothers with Borderline Personality Disorder* consists of 12 weekly sessions that are held by two female trainers experienced in working with patients with BPD. The development of the training was based on the concept of Dialectic Behavioral Therapy [23, 24]. Groups comprise four to eight mothers of children aged 0–6 and the group training can be applied in out- and inpatient treatment settings or assisted living facilities. It is important to note that the training does not substitute an individual psychotherapy focusing on the borderline symptomatology of the mother. It should be ensured that participants either already have completed or are still attending an individual therapy for their BPD.

Each session is divided into two parts, the first part focuses on the discussion of participants’ homework, in the second part a new topic is introduced. In each session a short leaflet about the topic is provided and work sheets with homework assignments are distributed. Each participant receives a folder for the handouts and information leaflets. The program includes the following modules. Except for *stress and stress management*, each module is delivered in one session:

### Psychoeducation

Psychoeducation is critical to the understanding of the impact of maternal borderline symptomatology on child development. In the first session, participants receive information about and are confronted with the risk associated with borderline-specific behaviors such as impulsivity, instability, loss of control, or self-harm for the cognitive and emotional development of their children. Participants are validated for their difficulties and encouraged for their participation in the group program while at the same time the necessity of changing and working on their problematic behavioral patterns is emphasized.

### Mindfulness

Short, regular mindfulness exercises for the mothers are incorporated into the group sessions. The main goal is to point to the importance of mindfulness in the interaction with children to better perceive and understand the needs of the children and to gain a greater control over own dysfunctional behavior impulses.

### Children’s basic needs

Mothers with BPD often show a lack of knowledge about the basic (emotional) needs (e.g., to feel safe) of children. The third session provides an overview regarding children’s needs, and the women are encouraged to assess their own competences to satisfy these needs.

### Stress and stress management

In two sessions the development and maintenance of stress as well as strategies to better cope with individual stressors are dealt with. For each mother, difficult situations in parenting are dealt with.

### Structure and flexibility

Mothers with BPD tend to show inconsistencies in the organization of the daily routine with their children. Therefore, this session aims to clarify the importance of rules and rituals and to better differentiate between fixed rules and flexible structures.

### Dealing with conflicts

Main goal of this 7th session is to promote a non-violent and solution-oriented management of mother-child conflicts. Here, role plays are particularly important to develop new ideas and ways to cope with conflicts.

### Dealing with emotions

Individuals with BPD often have difficulties in perceiving and regulating their emotions. In motherhood, women additionally need to comprehend their children’s emotions and needs. To better understand and handle intense maternal and infant emotions is the task of this session.

### The body

Women with BPD often report a lack of sensitivity for their own body and its needs. Additionally, they often aren’t familiar with the importance of the body and the body language when communicating with their children (e.g., kneeling beside the child while speaking vs. speaking from “above”). This session therefore aims to enhance the understanding of physical functions and reactions as well as to enhance the empathy for children’s needs for body care.

### Basic assumptions about parenting

Dysfunctional cognitions and assumptions about parenting (e.g., “a good mother never fails”) might constitute a core problem of mothers with BPD. The identification and modification of these assumptions into realistic views are the main goals of the 10th session.

### Self-care

Whereas all previous sessions mainly focus on children’s needs and the interaction with infants, the second to last session focuses on developing strategies to enable better maternal self-care. The importance of self-care for the emotional stability and satisfaction of the mother is emphasized.

The last session summarizes the content of the training and outlines the individual changes in the participants’ perception and parental behavior.

Each session starts with a short mindfulness practice. Subsequently, homework assignments of the previous session are discussed to facilitate learning and implementation of newly learned behaviors into daily life. After a break, the new subject is introduced followed by a specific exercise (e.g., role-play).

## Methods

During the pilot stage, the program was changed slightly (e.g., order of sessions) according to the feedback of the trainers and the group members.

### Participants

The participants of 4 groups (*n* = 15) were recruited via assisted living (Prowo e.V.) and a private psychotherapy practice. Participants’ age ranged from 20 to 42 years (M = 30.2). Two of the mothers had 3 children, one had 2 children, and the others had one child. Of the children 86% were under 4 years old, 46% were girls. With regard to the educational background, 66% of the mothers finished middle school, three had a university degree. Seven participants were staying in an assisted living facility, 8 were living at home. All mothers lived with their children. Seven women had a partner, 8 were single. All participants either had completed or were still in outpatient DBT therapy for their borderline symptoms.

### Inclusion and exclusion criteria

All participants had a one-on-one interview with one of the trainers before the start of the program. First of all, the trainer checked if the acute welfare of the child was endangered. In this case participation was not indicated. Additional exclusion criteria were current substance use or psychotic disorders. The BPD diagnosis was assured via clinicians’ diagnosis.

All participating mothers gave their written consent for data analyzation and publication. The authors assert that all procedures contributing to this work comply with the ethical standards of the relevant national and institutional committees on human experimentation and with the Helsinki Declaration of 1975, as revised in 2008.

### Procedure

From 2013 to 2015, three group trainings with 4 and one group training with 3 participants were conducted. Two female trainers experienced in working with patients with BPD instructed the groups. In each case at least one of the trainers had own children. Three groups took place in an assisted living home, one in a private practice. The sessions were held weekly. Questionnaires were completed in the first session (BDI, BSI, QTF, and PSS) and after the last session (open feedback). In the last session, participants were also asked to give written feedback regarding the complete training program (“What was especially helpful?”, “What was less helpful?”, “Which changes did occur due to the training?”, and “Do you have any further comments or suggestions for improvement?”). The answers to the open questions about the training program are summarized in Table 1. Trainers provided feedback in an intervision session after the training.

**Table 1:** Participants’ feedback

Summary of *n* = 15 written feedbacks
What was especially helpful?
− Role play (because of the specific situations) − The case examples, to talk about taboos − Examples for concrete parenting behaviors provided in role plays − That things were put in a nutshell, not too much homework − The exchange between the mothers − The informative teamwork in a small group − The “togetherness” and the help, to not feel being left alone anymore − To hear the “extremes” of others; the thought, it could have hit me harder − The appreciative style − New knowledge to cope with own feelings and to engage in mindfulness − Homework: it is hard to do homework assignments on a regular basis, but only by doing things in a daily routine is it possible to change/learn things. − I am glad I could participate as there is not much help for mothers like me − I had the feeling of trust and was able to show my weak points. − It was very helpful to reflect my own behavior and to get feedback from the other participants, to learn from the difficulties of others.
What could be improved?
− Too short − A few of the mindfulness exercises − I was embarrassed during the role play − Too much to read − I didn’t understand the self-care, was too complicated − Sometimes I felt overwhelmed and under pressure
Which changes occurred due to the training?
− I freak out less when my daughter escapes − I have less freak-outs and can better cope with difficult situations; my son registers the changes − Situation with brushing my kid’s teeth is much better − I am now able to assert myself in a consequent but compromising way − I can better cope with stress, play more with my son, and manage daily routine under less pressure. − I am more consequent, calmer, even when my child is aggressive; I feel less pressure to be perfect − Better coping with feelings and mindfulness − I am calmer and more balanced with my child − I can now detect difficult situations in time, and then I try to remain calm and try to support my child. − When my son cries I am not as stressed and annoyed anymore. − I yell less at my children. I am more reflected and mind the feelings of my child. I am less devaluating, more consequent and caring. − I am more aware of the difficulties in raising my child. And more aware of myself. My child benefits immediately from the things I learned. − I stopped telling my son that he as a person is annoying. It is only his behavior. − Not only me but also my child feels better.
Do you have any further comments or suggestions for improvement?
− Good program, interesting and difficult because of extreme insights about my own behavior. − Exhausting, sometimes sad but also gave strength. − The sessions where good, sometimes a bit difficult to adapt for a baby. − Everything was very interesting and important, but the training was too short. − I learned more in these 12 sessions than in 6 months of therapy. It was a pleasant group and everything was explained well. The information was very easy to implement as it was linked to the child. − Very informative, the training helped me to be more relaxed with the whole thing of “being a mother”: I feel much better than before and would like to participate again. − I was satisfied and hope that other mothers will benefit from the training − I learned a lot about myself and my relationship to my child. − I learned a lot that I can actually apply. − One session including older children and explaining to them what the problems of mothers with BPD are would be helpful − More time for each session; maybe each session twice

### Measures

The Brief Symptom Inventory (BSI; [25], German version: [26]) was applied to assess general psychopathology. With 53 items rated on a 5-point Likert-scale ranging from 0–4, the suffering from various symptoms during the last 7 days is evaluated. In total nine subscales assess somatization, obsessive-compulsive, interpersonal sensitivity, depression, anxiety, hostility, phobic anxiety, paranoid ideation, and psychoticism. All scales can be summarized to capture global psychological distress (Global Severity Index, GSI), evaluated via the overall mean score. We only used the GSI, where the internal consistency is high (α = .92; [26]).

The Beck Depression Inventory-II (BDI-II; [27], German version: [28]) measures the severity of self-reported depression. It consists of 21 items, rated on a 4-point (0 to 3) Likert-scale. The total score is obtained by summing the ratings for all items. Internal consistency is high (α > .84; [29]).

The Questionnaire of Thoughts and Feelings (QTF; [30]) assesses feelings, strategic cognitions and assumptions characteristic of BPD. The short version [31] consists of 14 items representing borderline-specific statements (e.g., “The way I am is unacceptable”). The total QTF score is obtained by calculating the mean score, which ranges between 1 and 5, indicating the self-reported level of borderline-specific cognitions. Internal consistency is excellent (α = .96; [31]).

The Parental Stress Scale (PSS; [32]) consists of 18 items rated on a 5-point Likert-scale (1 = strongly disagree … 5 = strongly agree), assessing subjective burden due to the role as a parent (e.g., “*I sometimes worry whether I am doing enough for my child(ren)”; “The major source of stress in my life is my child(ren)*”).

## Results

### Open feedback

Overall, the training received positive evaluations. Participants regarded role plays as particularly helpful, as well as the possibility to exchange with other mothers with BPD, to speak about taboos, to gain new knowledge and also to engage in homework (see Table 1). All participants wished to have more time for each topic and suggested either to do two sessions for every topic or to have the possibility to repeat the whole training. After the training mothers reported to be better able to cope with feelings and with stress, to feel less tension in the interaction with their children and to be calmer.

From the trainers’ viewpoint, a positive, appreciating attitude towards the participants was especially helpful. Mostly, the atmosphere was lively, open-minded and constructive. Situations in which mothers aggressively revealed a hostile and negative attitude towards their children were extremely challenging. In these situations, it was useful to focus on the particular goals of the current session as well as to avoid an escalation between the participants. In conclusion, the trainers were impressed by the motivation and gratitude of the participants and by the changes in their behavior and attitudes towards parenting.

### Statistics

The mean values of the PSS before the training (*M* = 48.27, *SD* = 7.1) indicated a high level of perceived stress compared to a non-clinical reference value of *M* = 37.18 [33] (*t (181) = 5.34, p < .001*). Mean value of the QTF (*M* = 2.69, *SD* = .88) was lower than expected for a borderline sample but comparable to a clinical control group [31]. The mean score for the BDI-II (*M* = 23.27, *SD* = 12.67) represents moderate depressive symptoms [27]. The score for the BSI (*M* = 1.15, *SD* = .51) was similar to a group of outpatients with various disorders [34].

## Discussion

First of all, the newly developed group training for mothers with BPD was successfully conducted in two different settings. The schedule and time frame of the program proved to be reasonable in both, the assisted living and the private practice setting. In the assisted living setting, the group training was easily added to the existing program for the mothers. Sometimes mothers missed a group session due to the lack of child care. As a consequence, in following sessions, childcare was provided if needed to ensure that mothers could attend the sessions.

The group was very well accepted by the participants. After the training all participants reported that they benefitted from the covered contents in terms of better understanding their children and reacting more appropriately to their own and to the children’s emotions and impulsive behaviors. The following examples may illustrate their responses: “I am more consequent, calmer, even when my child is aggressive; I feel less pressure to be perfect”, “I can now detect difficult situations in time, and then I try to remain calm and try to support my child”. It should be noted that some participants wanted to have more time for each of the topics and wished to repeat the training.

It was obvious that mothers with BPD are in need of help regarding their parenting skills and at the same time were very thankful for the provided assistance. A mother answered the question “What was especially helpful?” with “The *togetherness* and the help, to not feel being left alone anymore”. Most of the participating women reported concrete and observable changes in their own behavior and the consequences for their offspring (e.g., “When my son cries I am not as stressed and annoyed anymore”, “I have fewer freak-outs and can better cope with difficult situations; my son registers the changes”, “Not only I but also my child feels better”).

These first four groups were held in order to test the practicability and acceptance of the program. Overall four different group trainers led the groups. Three of the trainers were involved in developing the program, one was very experienced in working with mothers with BPD.

The mean level of perceived parental stress assessed via the PSS was high compared to a non-clinical sample [32]. In comparison to another group of parents with mental disorders (*M* = 43.35, [33]) the level of the current sample was also high. When looking at the items of the PSS it becomes obvious that the questionnaire assesses general assumptions and attitudes toward the own parental role (e.g. “*Caring for my child(ren) sometimes takes more time and energy than I have to give*” or “*I feel close to my child(ren)*”) rather than specific parental behavior or stress. Participating mothers reported borderline-specific cognitions comparable to patients with BPD but at a somewhat lower level, probably reflecting a successfully continuous or completed outpatient DBT [35]. Their general psychological distress was also still in the clinical range. This indicates that mothers with varying degrees of symptom burden may benefit from the program.

This group training is designed as addition to other treatments for the mothers. Psychotherapy for BPD focuses on the well-being of the parent. Usually there is not enough room to discuss parenting issues. At the same time there is an urgent need to address these problems as indicated by the feedback of the mothers. The main objective of the program is to interrupt the transmission of emotion-dysregulation from mother to child.

## Conclusion

There are many issues that still need to be addressed: The efficacy and effectiveness of the program should be evaluated in a large randomized controlled trial. The effects of the training on maternal parenting skills and attitudes, mother-child interaction patterns, psychological distress as well as children’s development and psychopathology should be analyzed. Additionally, the interaction between mothers and their offspring should be systematically observed and analyzed both before and after the training.

After conducting the first groups, writing the final version of the manual, and talking to trainers and mothers, we are convinced that *Parenting Skills for Mothers with Borderline Personality Disord*er is a promising program for mothers with BPD.

## Abbreviations

ADHD: Attention Deficit/Hyperactivity Disorder
BDI-II: Beck Depression Inventory II
BPD: Borderline Personality Disorder
BSI: Brief Symptom Inventory
DBT: Dialectical Behavior Therapy
PSS: Parental Stress Scale
QTF: Questionnaire of Thoughts and Feelings
